# Supplementary material for: Colistin Resistance Mechanisms in Human and Veterinary Klebsiella pneumoniae Isolates
Source: Antibiotics (Basel). 2022 Nov 21;11(11):1672. doi: 10.3390/antibiotics11111672 (PMC9686735; doi:10.3390/antibiotics11111672)
Supplement: Supplementary file 1 [file antibiotics-11-01672-s001.zip › antibiotics-2008465-supplementary.pdf]

Supporting Information

Colistin Resistance Mechanisms in Human and Veterinary *Klebsiella pneumoniae* Isolates

Manuela Tietgen [1,2,†], Lisa Sedlaczek [1,†], Paul G. Higgins [3], Heike Kaspar [4], Christa Ewers [5] and Stephan Göttig [1,*]

[1] Institute of Medical Microbiology and Infection Control, Hospital of the Johann Wolfgang von Goethe University, D-60596 Frankfurt am Main, Germany
[2] University Center of Competence for Infection Control of the State of Hesse, D-60596 Frankfurt am Main, Germany
[3] Institute for Medical Microbiology, Immunology and Hygiene, Faculty of Medicine and University Hospital Cologne, University of Cologne, D-50935 Cologne, Germany
[4] Federal Office of Consumer Protection and Food Safety, D-10117 Berlin, Germany
[5] Institute of Hygiene and Infectious Diseases of Animals, Justus Liebig University Giessen, D-35392 Giessen, Germany
* Correspondence: stephan.goettig@kgu.de
† These authors contributed equally to this work.

Supplementary Table S1. Primers used for detection and Sanger sequencing of beta-lactamase genes and mutations in the genes *mgrB*, *pmrAB* and *phoPQ.*

Name	Sequence (5'→3')	Gene	Size [bp]	Annealing temperature [°C]	Ref.
**CTX-M_F**	TTAGGAARTGTGCCGCTGYA	*bla*$_{CTX\text{-}M\text{-}1\text{-}like}$	688	60	11
**CTX-M_R**	CGATATCGTTGGTGGTRCCAT				
**SHV_schles_F**	TTTATCGGCCYTCACTCAAGG	*bla*$_{SHV}$	776	50	12
**SHV_schles_R**	AGATAAATCACCACAATGCGC				
***mgrB*_can_f**	AAGGCGTTCATTCTACCAC	*mgrB*	253	56	9
***mgrB*_can_R**	TTAAGAAGGCCGTGCTATCC				
***mgrB*_new_1F**	TTTGTATGATCCCTGGCGTGA	*mgrB*	269	56	This study
***mgrB*_new_1R**	AATAGTGCAAATGCCGCTGA				
***mgrB*_new_2F**	ACTGATAGTCATCATAGCAG	*mgrB*	344	56	This study
***mgrB*_new_2R**	AGCAGCGCTTGCAGCAGCTC				
***pmrA*_F**	CTCCACATGTTTCTGGTTGT	*pmrA*	960	58	This study
***pmrA*_R**	TCGCAGGATAATCTGTTCTC				
***pmrB*_F**	TTCTCTTATCGTCCTGCTTG	*pmrB*	1411	58	This study
***pmrB*_R**	CAATACCCTGGAAGTGCATA				
***phoP*_F**	GCAGGTGTCTGACAGGGATT	*phoP*	1467	58	This study
***phoP*_R**	AGGCTGAATACCCACAGGAC				
***phoQ*_F**	ATGACAGCGGGAAGATATGC	*phoQ*	672	58	This study
***phoQ*_R**	GAGCGTCAGACTACTATCGA				

Supplementary Table S2. Presence of antibiotic resistance genes in clinical *Klebsiella pneumoniae* isolates as determined by ResFinder (https://cge.food.dtu.dk/services/ResFinder/). The selected threshold for identification was 98% with a minimum length of 80%. All isolates harboured *oqxA, oqxB* and *fosA*.

	**Veterinary isolates**						**Human isolates**											
**Antibiotic class**	**IHIT27665**	**IHIT27662**	**IHIT27663**	**IHIT27664**	**IHIT32358**	**IHIT33535**	**KP_03**	**KP_53**	**KP_54**	**KP_1153**	**KP_1200**	**KP_1377**	**KP_1585**	**KP_1710**	**KP_1954**	**KP_2442**	**KP_3405**	**KP_3996**
**Beta-lactam (*bla*$_X$)[b]**	TEM-1B, SHV-28, **CTX-M-1**	SHV-27-like	SHV-1	SHV-36	SHV-28	TEM-1B, SHV-28, **CTX-M-15**	SHV-1, OXA-1	TEM-1B, SHV-1, OXA-1, **CTX-M-15**	SHV-11, OXA-1, DHA-1	TEM-1B, SHV-11, **CTX-M-15**	TEM-1B, **SHV-27**, OXA-1, **CTX-M-15**	TEM-1A, **SHV-12**, OXA-9, **KPC-2**	TEM-1B, SHV-11, OXA-1, **CTX-M-15**	TEM-1B, SHV-11, OXA-1, **CTX-M-15**	TEM-1A, OXA-9, **KPC-2**	TEM-1B, SHV-28, OXA-1, **CTX-M-15**	TEM-1A, SHV-28, OXA-1, **CTX-M-15**	SHV-1, **KPC-3**
**Aminoglycoside**	*aph(4)-Ia, aac(3)-IV*	-	-	-	*strA, strB, aadA2*	*aac(3)-IIa, strA, strB*	*aac(6’)Ib-cr, aac(3)-IIa, strA, aadA2*	*aadA2, aac(6’)Ib-cr*	*aac(6’)Ib-cr, aph(3’)-Ia*	*aac(6’)-Ib, aph(6)-Id, aph(3’’)-Ib*	*aac(3)-IIa, aac(6’)Ib-cr, aph(3’)-Ia, aadA2*	*aph(3’)-Ia, aac(6’)-Ib, aadA2*	*aadA2, aac(6’)Ib-cr, aac(3)-IId, aph(3’)-Ia*	*aac(6’)Ib-cr, aadA2*	*aph(3’)-Ia, strA, aadA2, aac(6’)-Ib*	*aac(6’)Ib-cr, aac(3)-IIa, aph(3’)-Ia, aadA1, aadA2*	*aac(3)-IId, aac(6’)Ib-cr*	*armA*
**Fluoroquinolone**	-	-	-	-	-	*qnrB1*	*aac(6’)Ib-cr*	*aac(6’)Ib-cr*	*aac(6’)Ib-cr, qnrB4*	*aac(6’)Ib-cr*	*aac(6’)Ib-cr*	*aac(6’)Ib-cr*	*aac(6’)Ib-cr*	*aac(6’)Ib-cr, qnrB4*	*aac(6’)Ib-cr, qnrB4*	*aac(6’)Ib-cr*	*aac(6’)Ib-cr*	-
**Trimethoprim**	-	-	-	-	*dfrA12*	*dfrA14*	*dfrA12*	*dfrA12*	-	*dfrA12*	*dfrA12*	*dfrA12*	*dfrA30*	*dfrA1*	*dfrA14, dfrA12*	*dfrA12*	*dfrA14, dfrA17*	-
**Sulphonamide**	-	-	-	-	-	*sul2*	*sul1, sul2*	*sul1*	*sul1*	*sul2*	*sul1*	*sul1*	*sul1*	*sul1*	*sul1, sul2*	*sul1, sul3*	-	-
**Tetracycline**	-	-	-	-	*tet(A)*	*tet(A)*	*tet(A)*	*tet(A)*	-	*tet(A)*	*tet(A)*	-	*tet(D)*	*tet(A)*	-	*tet(A)*	-	-
**MLS[a]**	*mph(A), mph(B)*	-	-	-	-	-	*mph(A)*	*mph(A)*	*mph(A)*	*mph(A)*	*mph(A)*	-	*mph(A)*	*mph(A)*	*mph(A)*	*mph(A)*	-	*msr(E), mph(E)*
**Phenicol**	*floR*	-	-	-	*catA1*	-	*catA1, catB4*	*catB4*	*catB3*	*catA2*	*catB4*	*catA1*	*catA1 catB4*	*catA1, catB4*	*catA1*	*catA1, catA4, floR, cmlA1*	*catB4*	-

[a]**MLS,** Macrolide-lincosamide-streptogramin B; [b] ESBLs and carbapenemases are indicated in bold.

Supplementary Table S3. Minimum inhibitory concentrations (MICs) for clinical *Klebsiella pneumoniae* isolates. All MICs were determined by antibiotic gradient tests unless otherwise indicated.

	Veterinary isolates						Human isolates											
**Antibiotic agent**	**IHIT27662**	**IHIT27663**	**IHIT27664**	**IHIT27665**	**IHIT32358**	**IHIT33535**	**KP_03**	**KP_53**	**KP_54**	**KP_1153**	**KP_1200**	**KP_1377**	**KP_1585**	**KP_1710**	**KP_1954**	**KP_2442**	**KP_3405**	**KP_3996**
**Ampicillin**	128	64	64	>256	>256	>256	>256	>256	>256	>256	>256	>256	>256	>256	>256	>256	>256	>256
**Ampicillin/ sulbactam[a]**	2	2	2	>256	>256	16	>256	>256	>256	>256	>256	>256	>256	>256	>256	>256	>256	>256
**Piperacillin**	2	8	8	>256	>256	>256	>256	>256	>256	>256	>256	>256	>256	>256	>256	>256	>256	>256
**Cefuroxime**	4	4	2	>256	4	>256	>256	>256	>256	>256	>256	>256	>256	>256	>256	>256	>256	>256
**Cefepime[b]**	0.064	0.032	0.032	16	0.125	2	32	>256	8	>256	>64	>64	>256	48	>256	>64	>256	>256
**Ertapenem**	0.016	0.016	0.016	8	0.032	0.064	0.125	>32	>32	>32	12	32	>32	0.125	>32	0.25	>32	>32
**Gentamicin**	0.5	0.5	0.5	8	1	32	32	2	1	2	>256	2	>256	1	2	16	16	>256
**Levofloxacin[b]**	≤0.125	≤0.125	≤0.125	4	>8	>8	1	>32	>32	>32	>32	>32	>32	>32	>32	>32	>32	>32
**Tigecycline**	0.5	0.5	0.5	4	2	1	0.5	2	0.5	1	8	1	2	2	4	2	2	2
**Colistin[c]**	8	8	8	8	4	2	8	8	8	4	4	4	4	8	16	2	2	2
**Colistin[d]**	24	12	24	24	12	32	24	48	256	256	6	12	16	256	256	6	8	12
**Colistin[e]**	>16	>16	>16	>16	>16	>16	8	>16	>16	>16	8	>16	>16	>16	>16	8	>16	8

[a]Sulbactam was used at a fixed concentration of 4 mg/L as recommended by EUCAST.
[b]MICs were determined by either antibiotic gradient test or VITEK® 2 analysis.
[c]MICs were determined using ETEST® and Mueller-Hinton agar (Oxoid).
[d]MICs were determined using ETEST® and Mueller-Hinton E agar (bioMérieux).
[e]MICs were determined by VITEK® 2 analysis.

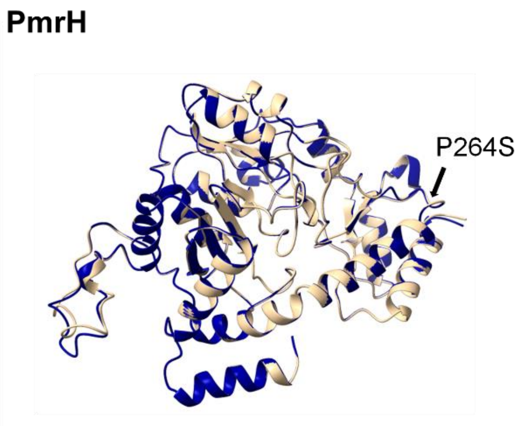

Figure S1 Superposition of PmrH of *K. pneumoniae* MGH78578 in blue (accession number WP_004889389.1) and PmrH of KP_03 in beige. Amino acid substitution P264S is marked by an arrow.

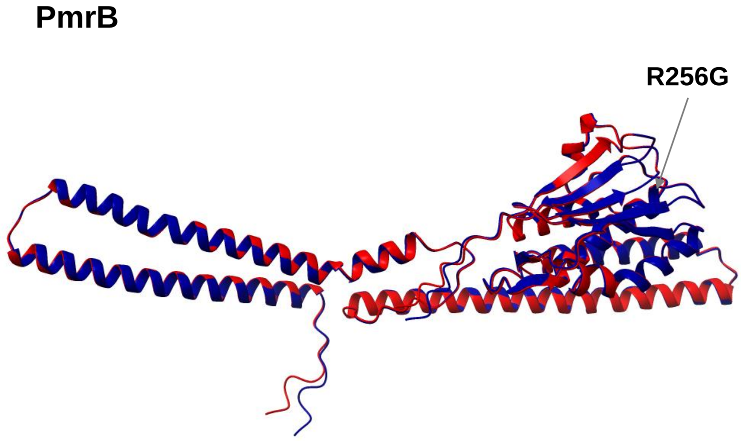

Figure S2 Superposition of PmrB of *K. pneumoniae* MGH78578 in blue and PmrB (R256G) of IHIT27665 in red. The arrow marks amino acid substitution R256G.

PmrB

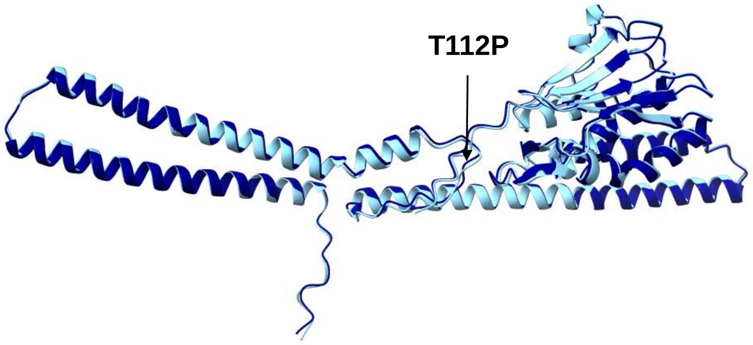

Figure S3 Superposition of PmrB of *K. pneumoniae* MGH78578 in dark blue and PmrB (T112P) of KP_54 (light blue). The arrow marks amino acid substitution T112P.
